# Does Time-to-Chemotherapy after Primary Complete Macroscopic Cytoreductive Surgery Influence Prognosis for Patients with Epithelial Ovarian Cancer? A Study of the FRANCOGYN Group

**DOI:** 10.3390/jcm10051058

**Published:** 2021-03-04

**Authors:** Grégoire Rocher, Thomas Gaillard, Catherine Uzan, Pierre Collinet, Pierre-Adrien Bolze, Marcos Ballester, Sofiane Bendifallah, Lobna Ouldamer, Cyril Touboul, Cyrille Huchon, Vincent Lavoue, Yohann Dabi, Cherif Akladios, Charles Coutant, Emilie Raimond, Alexandre Bricou, Geoffroy Canlorbe, Henri Azaïs

**Affiliations:** 1 Assistance Publique des Hôpitaux de Paris (AP-HP), Department of Gynecological and Breast Surgery and Oncology, Pitié-Salpêtrière University Hospital, 75013 Paris, France; gregoire.rocher@gmail.com (G.R.); tomas.gaillard@gmail.com (T.G.); catherine.uzan@aphp.fr (C.U.); geoffroy.canlorbe@aphp.fr (G.C.); 2 INSERM UMR_S_938, Cancer Biology and Therapeutics, Centre de Recherche Saint-Antoine (CRSA), Sorbonne University, 75020 Paris, France; 3 Institut Universitaire de Cancérologie (IUC), 75020 Paris, France; 4 Department of Gynecologic Surgery, Jeanne de Flandre Hospital, CHU de Lille, 59120 Loos, France; pierre.collinet@chru-lille.fr; 5 Gynecological Surgery Service, CHU Lyon-Sud, 69000 Lyon, France; pierre-adrien.bolze@chu-lyon.fr; 6 Department of Gynecologic and Breast Surgery, Groupe Hospitalier Diaconesses Croix Saint-Simon, 75012 Paris, France; mballester@hopital-dcss.org; 7 Department of Gynaecology and Obstetrics, Tenon University Hospital, Assistance Publique des Hôpitaux de Paris (AP-HP), University Pierre and Marie Curie, Paris 6, 75020 Paris, France; sofiane.bendifallah@aphp.fr (S.B.); cyril.touboul@aphp.fr (C.T.); 8 Department of Obstetrics and Gynaecology, Centre Hospitalier Régional Universitaire de Tours, Hôpital Bretonneau, 37000 Tours, France; l.ouldamer@chu-tours.fr; 9 Department of Obstetrics and Gynecology, Poissy-St Germain Hospital, 78300 Poissy, France; cyrille.huchon@aphp.fr; 10 Service de Gynécologie, CHU de Rennes, Université de Rennes 1, 35033 Rennes, France; Vincent.lavoue@chu-rennes.fr; 11 Department of Gynecology and Obstetrics, Université de Médecine Paris Est Créteil (Paris XII), Centre Hospitalier Intercommunal, 94000 Créteil, France; yohann.dabi@aphp.fr; 12 Department of Gynecology and Obstetrics, CHU, 67000 Strasbourg, France; cherif.akladios@gmail.com; 13 Department of Surgical Oncology, Georges-François Leclerc Cancer Center, 21000 Dijon, France; ccoutant@cgfl.fr; 14 Department of Obstetrics and Gynaecology, Institute Alix de Champagne University Hospital, 51100 Reims, France; emilie_raimond@hotmail.com; 15 Department of Obstetrics, Gynecology and Reproductive Medicine, CH Jean Verdier, Assistance Publique-Hôpitaux de Paris (AP-HP), 93140 Bondy, France; alex.bricou@gmail.com

**Keywords:** epithelial ovarian cancer, chemotherapy, prognosis

## Abstract

To determine if the time-to-chemotherapy (TTC) after primary macroscopic complete cytoreductive surgery (CRS) influences recurrence-free survival (RFS) and overall survival (OS) in patients with epithelial ovarian cancer (EOC). We conducted an observational multicenter retrospective cohort analysis of women with EOC treated from September 2006 to November 2016 in nine institutions in France (FRANCOGYN research group) with maintained EOC databases. We included women with EOC (all FIGO stages) who underwent primary complete macroscopic CRS prior to platinum-based adjuvant chemotherapy. Two hundred thirty-three patients were included: 73 (31.3%) in the early-stage group (ESG) (FIGO I-II), and 160 (68.7%) in the advanced-stage group (ASG) (FIGO III-IV). Median TTC was 43 days (36–56). The median OS was 77.2 months (65.9–106.6). OS was lower in the ASG when TTC exceeded 8 weeks (70.5 vs. 59.3 months, *p* = 0.04). No impact on OS was found when TTC was below or above 6 weeks (78.5 and 66.8 months, respectively, *p* = 0.25). In the whole population, TTC had no impact on RFS or OS. None of the factors studied were associated with an increase in TTC. Chemotherapy should be initiated as soon as possible after CRS. A TTC greater than 8 weeks is associated with poorer OS in patients with advanced stage EOC.

## 1. Introduction

Epithelial ovarian cancer (EOC) represents 3.8% of all female cancers worldwide and is the fourth cause of death from cancer among women [1,2]. In France, 5183 women were diagnosed with EOC in 2018 [3]. The prognosis of EOC is poor with a 5-year overall survival (OS) rate of under 50%. It is usually diagnosed at an advanced stage (i.e., stage III–IV according to the 2014 International Federation of Gynecology and Obstetrics (FIGO) classification), mainly due to the lack of an effective screening method and unspecific symptoms [2,4,5,6,7]. Standard treatment for both early- and advanced-staged EOC consists of complete macroscopic cytoreductive surgery (CRS) followed by adjuvant platinum-based chemotherapy [2,4,8]. Despite the benefits of CRS, the procedure is associated with substantial perioperative morbidity, especially for women with advanced FIGO stage, which has been shown to delay the initiation of adjuvant chemotherapy in observational studies [9,10].

Time-to-chemotherapy (TTC) has been explored as a prognostic factor in terms of recurrence-free survival (RFS) and OS. Risk factors for increased TTC have already been identified [11,12] and include advanced age (over 65 years) [13], comorbidities, and geographic situations. The published studies show a significant decrease in RFS and OS when TTC exceeds 12 weeks [2,4] in women with advanced-stage EOC, and consequently recommend initiating chemotherapy within the first 30 to 42 days after CRS [2,6,10,14,15,16,17,18]. The aim of our study was to determine if the TTC after primary CRS influences prognosis (RFS and OS) in patients with EOC.

## 2. Method

### 2.1. Study Population

We conducted a multicenter retrospective cohort analysis of women with EOC treated from September 2006 to November 2016 in nine institutions in France (the FRANCOGYN research group) with maintained EOC databases. The study was approved by the ethics committee of the National College of French Obstetricians and Gynecologists (CEROG 2020-GYN-0802) and patients were duly informed about the study as required by French law.

The study population was women with EOC classified into two subgroups according to their stage as defined in the 2014 International Federation of Gynecology and Obstetrics (FIGO) classification [19]: Early-Stage Group (ESG) (FIGO I-II) and Advanced-Stage Group (ASG) (FIGO III-IV) [19,20]. TTC was defined as the time between primary CRS and the initiation of chemotherapy. We hypothesized that a delay in initiating chemotherapy after CRS could impact survival and chose to study two TTC thresholds: 6 weeks as defined in the French recommendations [2], and 8 weeks as it is commonly stated that a TTC over 6 weeks is associated with poorer outcomes. All women with EOC who had undergone primary complete macroscopic CRS were included. All patients received adjuvant therapy (6 cycles of adjuvant platinum-based chemotherapy: carboplatin AUC5, paclitaxel 175/m^2^, ±targeted therapy according to international guidelines at the time of management). Tumor histology was classified as serous, mucinous, endometrioid, or clear cell. Data about peri- and postoperative complications were not available. Patients treated by neoadjuvant chemotherapy or chemotherapy alone were excluded. Patients with ovarian borderline tumors and patients who underwent CRS with macroscopic residual tumor after surgery were excluded. The following clinical and pathologic variables were collected: age, comorbidities, parity, surgical procedure, stage according to the 2014 FIGO classification [19], final pathologic analysis, adjuvant therapies, date of recurrence, death, or latest news.

### 2.2. Endpoints

The endpoints of the study were RFS and OS, defined as the time between CRS and first progression or time between CRS and death, respectively. If none of the events had occurred at the time of last contact, patients were censored. Recurrence was determined on clinical signs or by imaging studies.

### 2.3. Statistical Analysis

Clinical characteristics were compared for univariate analysis by Chi-Square or Fisher Exact tests and by Student T-tests for quantitative variables as appropriate. All statistical tests used a significance level of 5%. The survival outcomes are presented using the Kaplan–Meier method, and compared by log-rank tests. The effect of variables on RFS were tested by the univariate cox regression model and the proportional hazards assumption was tested. A multivariate Cox model was then performed considering potentially confounding factors. Analyses were performed with R software, version 3.5.2 (R Core Team^®^ 2008 by the R Foundation for Statistical Computing, Vienna, Austria).

## 3. Results

### 3.1. Population Characteristics

**Figure 1:**
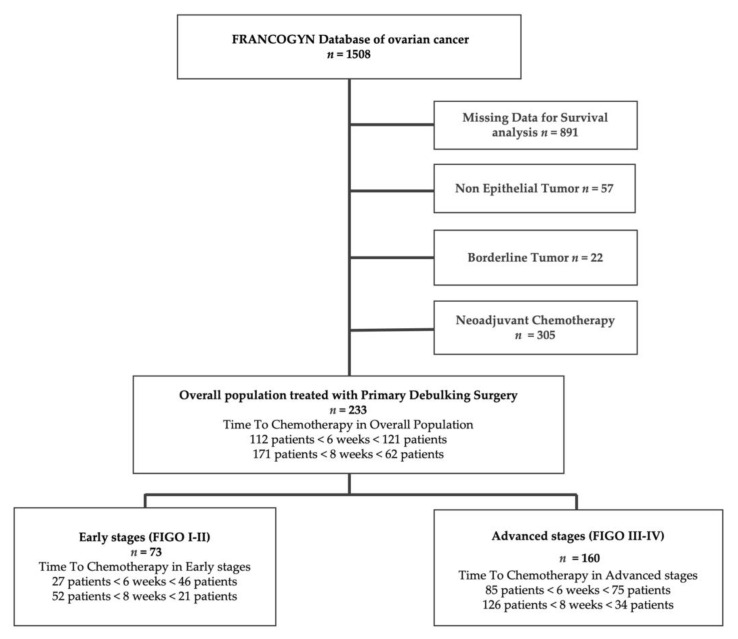
Study flowchart. FIGO: International Federation of Gynecology and Obstetrics classification.

Overall, 1508 women were treated for EOC in the nine study centers during the study period. Nine hundred and seventy patients were excluded (discordant or lack of data, borderline, or non-epithelial tumors). Three hundred and five patients received neoadjuvant chemotherapy and were also excluded (Figure 1). Two hundred thirty-three patients were thus retained for analysis: 73 (31.3%) with early-stage EOC (ESG) and 160 (68.7%) with advanced-stage (ASG).

The patient characteristics are summarized in Table 1 and Table 2. The median TTC was 43 (36–56) days, median RFS was 22 (42.5–1.51) months, and median OS was 77.2 (65.9–106.6) months. The median age at diagnosis was 59 (47–71) years. Most of the patients presented with advanced-stage EOC (*n* = 160; 68.7%) with stage III (60.5%) and stage IV (8.2%) tumors. All patients received standard platinum-based treatment for adjuvant chemotherapy, mainly six cycles of carboplatin and paclitaxel every 21 days. The most frequent type of recurrence was peritoneal carcinomatosis (65.5%), followed by lymph node recurrence (16.7%), and then visceral metastasis (9.4%). Seventy-three percent of the patients (171) received adjuvant chemotherapy within 8 weeks after CRS. The baseline characteristics (age, BMI, hormonal status, comorbidities, grade, and histologic type) did not differ in the populations of patients with a TTC under or over 6 and 8 weeks. The relative hazard of RFS plotted by TTC showed no relation between TTC and RFS regarding variable linearity. This analysis (with 95% confidence intervals) confirmed that relative hazard of RFS had no impact, regardless of the TTC duration.

In the ESG, the median TTC was 48 (36.0–51.3) days. The median follow-up time was 44.75 months. The baseline characteristics (age, BMI, hormonal status, comorbidities, grade, and histologic type) did not differ in the populations of patients with a TTC under or over 6 and 8 weeks (Tables S1 and S2). Tumor stage was mostly FIGO Stage 1. The most frequent type of recurrence was peritoneal carcinomatosis, as in the whole population.

In the ASG, the median TTC was 41 (11.2–70.7) days. Median follow-up time was 46 months. The baseline characteristics (age, BMI, hormonal status, comorbidities, grade, and histologic type) did not differ in the populations of patients with a TTC under or over 6 and 8 weeks (Tables S3 and S4). Most of the patients in the ASG had a FIGO stage III tumor. Only 41 patients in this group had a TTC over 8 weeks.

### 3.2. Survival Analysis

#### 3.2.1. Whole Population

There was no significant impact of TTC on RFS (HR = 1.03 (0.98–1.09) *p* = 0.23) in the whole population (Table S5). There was no significant difference in OS between patients with a TTC below or above 6 weeks (78.5 versus 66.8 months, respectively, *p* = 0.25). There was a non-significant trend in favor of a higher OS in patients with a TTC under 8 weeks as compared with those with a TTC over 8 weeks (78.5 and 60.1 months, respectively, *p* = 0.08) (Table 3). The Kaplan–Meier curves showed no significant difference in RFS or in OS between the patients whose TTC was below 6 and above 8 weeks (*p* = 0.64 for RFS and 0.22 for OS). None of the factors associated with surgical comorbidities were found to be associated with RFS (Table S5): diabetes (HR 1.61 (0.7–3.69) *p* = 0.26), hypertension (HR 1.2 (0.74–1.95) *p* = 0.46), or smoking (HR 2.07 (0.88–4.89) *p* = 0.09).

#### 3.2.2. Early-Stage Group

**Figure 2:**
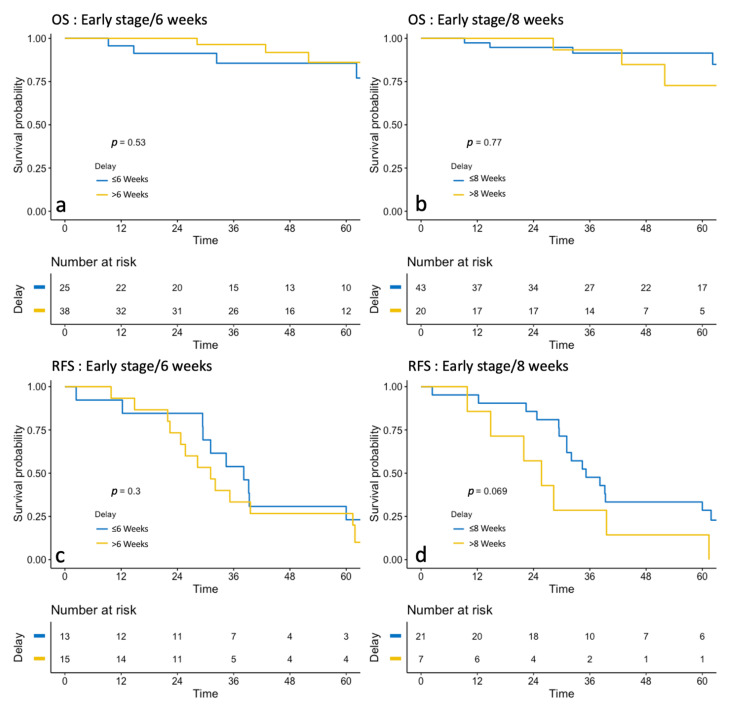
Survival analysis of Overall Survival (OS) (before and after 6 weeks: (**a**) before and after 6 weeks: (**b**) and Recurrence-free Survival (RFS) (before and after 6 weeks: (**c**), before and after 6 weeks: (**d**) in Early stages.

Medians of overall survival could not be presented for the ESG because fewer than 50% of this population presented the event during the follow-up period (Table 3). In the Kaplan–Meier analyses, TTC was not associated with a difference in RFS or OS according to whether the TTC was under or over 6 and 8 weeks (Figure 2). For RFS, no difference was found according to whether the TTC was under or over 6 (*p* = 0.3) or 8 weeks (*p* = 0.069). Likewise, TTC had no impact on OS, whether it was under or over 6 (*p* = 0.53) or 8 weeks (*p =* 0.77).

#### 3.2.3. Advanced-Stage Group

**Figure 3:**
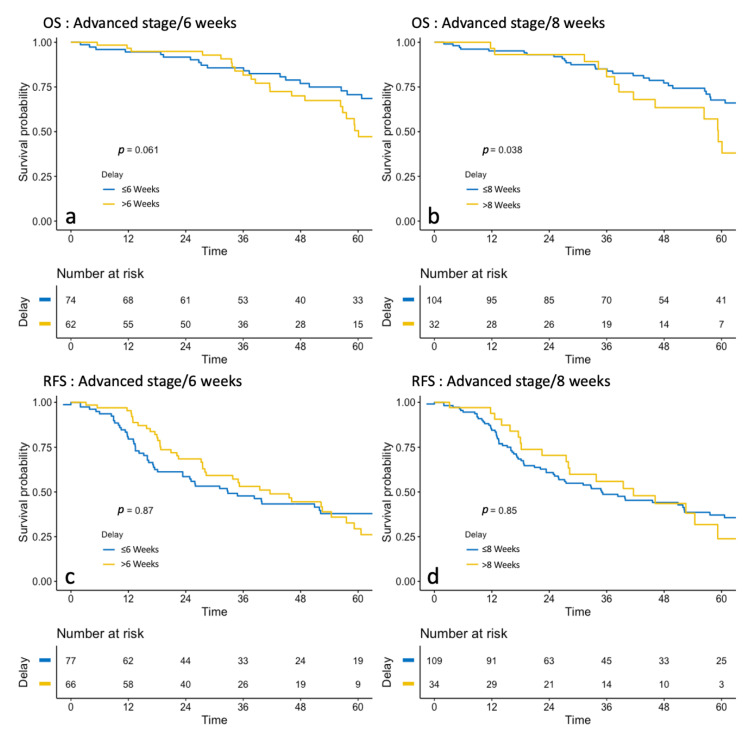
Survival analysis of Overall Survival (OS) (before and after 6 weeks: (**a**), before and after 6 weeks: (**b**) and Recurrence-free Survival (RFS) (before and after 6 weeks: (**c**), before and after 6 weeks: (**d**) in Advanced stages.

In the Kaplan–Meier analyses (Figure 3), no difference was found in RFS if TTC was under or over 6 (*p* = 0.087) or 8 weeks (*p* = 0.85). Regarding OS, there was no significant difference when TTC was under 6 or over 6 weeks (77.2 months versus 60.1 months, respectively, *p* = 0.06). When TTC was under 8 weeks, the median OS was 70.5 months compared with 59.3 months when it was over 8 weeks (*p* = 0.04) (Table 3).

### 3.3. Cox Univariate and Multivariate Analysis

Using univariate cox regression models (Table S5), none of the tested parameters had a significant impact on RFS (age, BMI, hormonal status, comorbidities, FIGO stage, grade, lymphovascular space involvement (LVSI) status, and histologic type). In the multivariate model, TTC was not associated with RFS (HR: 0.99 (0.91–1.09), *p =* 0.91) after adjustment on covariates. The multivariate cox regression model did not show a significant survival impact of TTC on RFS (HR: 1.03 (0.81–1.32), *p =* 0.79, Table S6).

## 4. Discussion

In this retrospective multicenter study, we assessed survival (RFS and OS) according to TTC after primary complete CRS in 233 patients with newly diagnosed EOC of all FIGO stages. With a 6-week cutoff, there was a non-significant trend in favor of a better OS and RFS in patients with a TTC below 6 weeks in the whole population and in the ASG. With an 8-week cutoff, there was a trend in favor of a higher OS in patients with a TTC below 8 weeks in the whole population (+8.4 months, *p* = 0.08), and a significantly higher OS in the ASG (70.5 vs. 59.3 months, *p* = 0.04).

We did not observe any significant impact of TTC on OS or RFS either in the whole population or in the ESG, whether it was under or over 6 or 8 weeks. We observed a median TTC of under 6 weeks in the ASG, but the average TTC was 43 days which is slightly higher than that presented in other studies [4,15,17,21]. In the ESG, the median TTC was 48 days, which is over the recommended 6 weeks, but did not have any impact on RFS or OS. In this multicenter retrospective study, the date of death is considered as solid data, but the date of recurrence could be less precise due to differences in diagnosis.

The impact of TTC on the prognosis of patients with EOC undergoing CRS has already been described. However, while studies published to date frequently reveal a trend towards an impact on cancer specific survival [5,6,12,14,15,16,17,22], significant results seem to be harder to demonstrate [18,21,22,23]. Some studies which did not find any relationship between TTC and survival [24] included patients with macroscopic residual disease. The optimal TTC would appear to be between 22 and 28 days [2,4,15,17,22] with a greater impact on OS after 6 weeks [2,10,18,21], but no benefit has been demonstrated with a TTC before 4 weeks [4,15]. Surgical complications influence the TTC, but also the OS independently. In 2012, Wright et al. showed that patients with more than two surgical complications were 31% more likely to die from ovarian cancer regardless of the effect of TTC [4]. They examined intra- and post-operative risk factors for delaying or omitting adjuvant chemotherapy after primary CRS among 3991 patients: 12% never received adjuvant chemotherapy, 24% received their first cycle of chemotherapy 6 weeks after surgery, and 4% 12 weeks after surgery. The risks factors for not receiving chemotherapy were an advanced age (over 65 years), medical comorbidities, patients with mucinous tumors, and patients with stage IV disease. Extended CRS and multiple postoperative complications were associated with a delay in receiving chemotherapy. No difference was found in OS between patients with a TTC under 6 weeks and a TTC between 6 and 12 weeks. However, OS was poorer for patients with a TTC of over 12 weeks. In 2013, Mahner et al. found that a longer TTC was associated with a trend towards earlier progression in patients with no residual tumor after surgery [18]: increasing TTC by 7 days resulted in an 8.7% increase in mortality risk for each week passed. Seagle et al. conducted a retrospective study of 45,001 patients registered in the National Cancer Database (USA) between 1998 and 2011 [15]. They observed that TTC was over 28 days for 58.1% of the patients and that women with a TTC of between 21 and 35 days experienced a 7% decreased HR for death as compared to those with a TTC of 36 days or more.

Due to the high rate of missing information in our database regarding dates of surgery or first cycle of adjuvant chemotherapy, only 15% of the patients were included. This somewhat limits the extrapolation of the results to the general population. In addition, the inclusion of low grade endometroid, mucinous, and clear cell tumors, which are less chemosensitive could be a limitation of our conclusions on the impact of TTC on RFS and OS. Furthermore, the database did not provide information about specific digestive resection, or about detailed postoperative complications. Neither did this retrospective study reflect current adjuvant therapies that may include PARP inhibitors [25] and bevacizumab as first-line maintenance [26]. Patients who underwent CRS with macroscopic residual tumor after surgery could have been a major prognosis bias in the OS analysis; they were excluded even if it limited the exportation of the study results in this population. Finally, most of the patients had pelvic and paraaortic lymphadenectomy, a procedure which may impact TTC but which is performed less frequently since its indications were restricted in 2017 [27].

Our observations show that the guidelines are largely respected, and that our data are concordant with previously publications: a TTC exceeding 8 weeks after CRS impacts OS in patients with advanced-stage EOC. These results are a conclusive validation of previous reports [9,15,18,28] and should encourage clinicians to closely follow-up women who are at a higher risk of delayed chemotherapy initiation.

## 5. Conclusions

Our results show a significant decrease in OS when TTC exceeds 8 weeks in the ASG. However, we observed a similar effect when TTC exceeded 6 weeks in the whole population and in patients with advanced stage EOC for PFS and OS. These data suggest that it is advisable to start chemotherapy as soon as possible after CRS and before 6 to 8 weeks. Our study results agree with those in the literature: they underline the absolute necessity of optimizing the time of surgery to avoid per- and postsurgical complications and allow a short TTC. Overall, women with EOC should be managed by an experienced gynecologic oncology surgical team in a certified center for cancer management [27,29].

## Figures and Tables

**Table 1:** Characteristics of the patients. Time-to-Chemotherapy (TTC) before and after 6 weeks.

Variables			≤6 Weeks	>6 Weeks	*p*
		*n* = 233	*n* = 116	*n* = 117	
Age (years)		59 (±12)	58 (±12)	60 (±12)	0.41
BMI (kg/m^2^)		24.58 (±4.88)	24.09 (±4.79)	25.08 (±4.94)	0.13
Parity		1.64 (±1.42)	1.798 (±1.52)	1.482 (±1.3)	0.1
Mutation	BRCA 1	12 (5.1%)	8 (6.8%)	13 (11.1%)	0.14
	BRCA 2	5 (2.1%)	1 (3.4%)	0	
Hypertension		42 (24.6%)	20 (22.5%)	22 (26.8%)	0.51
Diabetes		10 (6.2%)	6 (7.1%)	4 (5.2%)	0.85
Smoking		12 (8.5%)	5 (6.8%)	7 (10.1%)	0.48
Histologic type	Serous	155 (70.1%)	77 (74.5%)	80 (66.1%)	0.71
	Endometrioid	37 (16.7%)	15 (14.2%)	22 (19.1%)	
	Clear Cell	23 (10.4%)	10 (9.7%)	13 (10.7%)	
	Mucinous	6 (2.7%)	2 (1.9%)	4 (3.3%)	
Grade	1	21 (67.7%)	9 (75%)	12 (63.2%)	0.23
	2	7 (22.6%)	1 (8.3%)	6 (31.6%)	
	3	3 (9.7%)	2 (16.7%)	1 (5.3%)	
Lymphovascular space involvement	Yes	35 (43.8%)	22 (46.8%)	13 (39.4%)	0.51
Stage	Early	69 (29.6%)	89 (76.7%)	75 (64.1%)	0.035
	Advanced	164 (70.4%)	27 (23.3%)	42 (35.9%)	
FIGO Stage	I	56 (24%)	18 (15.5%)	38 (32.5%)	0.0092
	II	17 (7.2%)	11 (9.5%)	6 (5.1%)	
	III	141 (60.5%)	74 (63.8%)	67 (57.3%)	
	IV	19 (8.1%)	13 (11.2%)	6 (5.1%)	
Type of recurrence	Lymph node	16 (16.7%)	9 (17%)	7 (16.3%)	0.22
	Peritoneal Carcinomatosis	63 (65.6%)	37 (69.8%)	26 (60.5%)	
	Metastasis	9 (9.4%)	2 (3.8%)	7 (16.3%)	

FIGO: International Federation of Gynecology and Obstetrics.

**Table 2:** Characteristics of the patients. Time-to-Chemotherapy before and after 8 weeks.

Variables			≤8 weeks	>8 weeks	*p*
		*n* = 233	*n* = 175	*n* = 58	
Age (years)		59 (±12)	58 (±12)	60 (±12)	0.41
BMI (kg/m^2^)		24.58 (±4.88)	24.51 (±4.74)	24.79 (±5.34)	0.72
Parity		1.64 (±1.42)	1.758 (±1.48)	1.278 (±1.17)	0.031
Mutation	BRCA 1	12 (5.1%)	17 (9.7%)	4 (2.8%)	0.8
	BRCA 2	5 (2.1%)	1 (0.6%)	0	
Hypertension		42 (24.6%)	32 (25%)	10 (23.3%)	0.82
Diabetes		10 (6.2%)	8 (6.7%)	2 (4.8%)	1
Smoking		12 (8.5%)	8 (7.6%)	4 (10.8%)	0.8
Histologic type	Serous	155 (70.1%)	116 (70.7%)	39 (68.4%)	0.94
	Endometrioid	37 (16.7%)	27 (16.5%)	10 (17.5%)	
	Clear Cell	23 (10.4%)	17 (10.4%)	6 (10.5%)	
	Mucinous	6 (2.7%)	4 (2.4%)	2 (3.5%)	
Grade	1	21 (67.7%)	16 (72.7%)	5 (55.6%)	
	2	7 (22.6%)	4 (18.2%)	3 (33.3%)	
	3	3 (9.7%)	2 (9.1%)	1 (11.1%)	
Lymphovascular space involvement	Yes	35 (43.8%)	27 (45.8%)	8 (38.1%)	0.54
Stage	Early	69 (29.6%)	127 (72.6%)	37 (63.8%)	0.2
	Advanced	164 (70.4%)	48 (27.4%)	21 (36.2%)	
FIGO Stage	I	56 (24%)	36 (20.6%)	20 (34.5%)	0.068
	II	17 (7.2%)	16 (9.1%)	1 (1.7%)	
	III	141 (60.5%)	108 (61.7%)	33 (56.9%)	
	IV	19 (8.1%)	15 (8.6%)	4 (6.9%)	
Type of recurrence	Lymph node	16 (16.7%)	12 (16.2%)	4 (18.2%)	0.84
	Peritoneal Carcinomatosis	63 (65.6%)	47 (63.5%)	16 (72.7%)	
	Metastasis	9 (9.4%)	8 (10.8%)	1 (4.5%)	

FIGO: International Federation of Gynecology and Obstetrics.

**Table 3:** Overall Survival according to the TTC the whole population and Early- and Advanced-stage groups, when Time to Chemotherapy occurs before and after 6 and 8 weeks.

	Overall Survival. Median, Months		
**Time to Chemotherapy**	**TTC < 6 weeks**	**TTC > 6 Weeks**	***p***
Whole population	78.5	66.8	0.3
Early-stage group	NA	NA	
Advanced-stage group	77.2	60.1	0.06
Time to Chemotherapy	TTC < 8 weeks	TTC > 8 weeks	
Whole population	78.5	60.1	0.08
Early-stage group	NA	NA	
Advanced-stage group	70.5	59.3	0.04

## Data Availability

The data presented in this study are available on request from the corresponding author.

## Supplementary Materials

The following are available online at https://www.mdpi.com/2077-0383/10/5/1058/s1, Table S1: Characteristics of Patients in the early-stage group. Time-to-Chemotherapy before and after 6 weeks, Table S2: Characteristics of Patients in the early-stage group. Time-to-Chemotherapy before and after 8 weeks, Table S3: Characteristics of Patients in the advanced-stage group. Time-to-Chemotherapy before and after 6 weeks, Table S4: Characteristics of Patients in the advanced-stage group. Time-to-Chemotherapy before and after 8 weeks, Table S5: Univariate Cox regression model—Recurrence-free Survival, Table S6: Multivariate Cox regression model—Recurrence-free Survival.
