# Supplementary material for: Does Time-to-Chemotherapy after Primary Complete Macroscopic Cytoreductive Surgery Influence Prognosis for Patients with Epithelial Ovarian Cancer? A Study of the FRANCOGYN Group
Source: J Clin Med. 2021 Mar 4;10(5):1058. doi: 10.3390/jcm10051058 (PMC7961531; doi:10.3390/jcm10051058)
Supplement: Supplementary file 1 [file jcm-10-01058-s001.pdf]

SUPPLEMENTARY DATA

Table S1: Characteristics of Patients in the early-stage group. Time-to-Chemotherapy before and after 6 weeks

Variables	-	≤6 weeks (n = 27)	>6 weeks (n = 42)	p
Age (years)		58 (+/- 14)	58 (+/- 12)	0.93
BMI (Kg/m2)		24.51 (+/- 5.25)	25.84 (+/- 6.28)	0.38
Hormonal Status	Menopause	18 (69.2%)	29 (72.5%)	0.77
	Menopausal Hormone Therapy	4 (36.4%)	5 (25%)	0.8
Parity		1.2 (+/- 1.44)	1.211 (+/- 1.02)	0.97
Mutation	BRCA 1	1 (14.3%)	2 (22.2%)	0.76
	BRCA 2	NA	NA	
Hypertension		8 (36.4%)	7 (25%)	0.38
Diabetes		3 (15.8%)	1 (3.6%)	0.29
Smoking		2 (10%)	4 (13.3%)	1
Histologic type	Serous	10 (37%)	19 (45.2%)	0.52
	Endometrioid	8 (29.6%)	14 (33.3%)	
	Clear Cell	6 (22.2%)	8 (19%)	
	Mucinous	3 (11.1%)	1 (2.4%)	
Grade	1	5 (83.3%)	6 (50%)	0.19
	2	0	5 (41.7%)	
	3	1 (16.7%)	1 (8.3%)	
FIGO Stage	I	18 (66.7%)	38 (90.5%)	0.025
	IIA	9 (33.3%)	4 (9.5%)	
Type of recurrence	Lymph node	2 (18.2%)	1 (11.1%)	0.68
	Peritoneal Carcinomatosis	6 (54.5%)	6 (66.7%)	
	Metastasis	1 (9.1%)	2 (22.2%)	

Table S2: Characteristics of Patients in the early-stage group. Time-to-Chemotherapy before and after 8 weeks

Variables		≤8weeks (*n* = 48)	>8weeks (*n* = 21)	*p*
Age (years)		58 (+/- 12)	56 (+/- 14)	0.44
BMI (Kg/m2)		25.25 (+/- 5.91)	25.4 (+/- 5.93)	0.93
Hormonal Status	Menopause	33 (73.3%)	14 (66.7%)	0.58
	Menopausal Hormone Therapy	5 (23.8%)	4 (40%)	0.42
Parity		1.196 (+/- 1.24)	1.235 (+/- 1.09)	0.91
Mutation	BRCA 1	3 (30%)	0	0.33
	BRCA 2	NA	NA	
Hypertension		13 (36.1%)	2 (14.3%)	0.24
Diabetes		4 (12.1%)	0	0.3
Smoking		4 (11.8%)	2 (12.5%)	1
Histologic type	Serous	20 (41.7%)	9 (42.9%)	0.87
	Endometrioid	14 (29.2%)	8 (38.1%)	
	Clear Cell	11 (22.9%)	3 (14.3%)	
	Mucinous	3 (6.2%)	1 (4.8%)	
Grade	1	10 (71.4%)	1 (25%)	0.16
	2	3 (21.4%)	2 (50%)	
	3	1 (7.1%)	1 (25%)	
FIGO Stage	I	36 (75%)	20 (95.2%)	0.09
	IIA	12 (25%)	1 (4.8%)	
Type of recurrence	Lymph node	3 (20%)	0 (0%)	0.87
	Peritoneal Carcinomatosis	8 (53.3%)	4 (80%)	
	Metastasis	2 (13.3%)	1 (20%)	

Table S3: Characteristics of Patients in the advanced-stage group. Time-to-Chemotherapy before and after 6 weeks

Variables		≤6 weeks (*n* = 89)	>6 weeks (*n* = 75)	*p*
Age (years)		59 (+/- 12)	61 (+/- 11)	0.2
BMI (Kg/m2)		23.95 (+/- 4.67)	24.68 (+/- 4.05)	0.31
Hormonal Status	Menopause	63 (76.8%)	56 (78.9%)	0.76
	Menopausal Hormone Therapy	17 (33.3%)	14 (31.8%)	0.88
Parity		1.976 (+/- 1.5)	1.625 (+/- 1.42)	0.14
Mutation	BRCA 1	3 (13.6%)	9 (40.9%)	0.098
	BRCA 2	4 (18.2%)	1 (4.5%)	
Hypertension		12 (17.9%)	15 (27.8%)	0.2
Diabetes		3 (4.6%)	3 (6.1%)	1
Smoking		3 (5.7%)	3 (7.7%)	0.7
Histologic type	Serous	68 (86.1%)	58 (79.5%)	0.54
	Endometrioid	7 (8.9%)	8 (11%)	
	Clear Cell	4 (5.1%)	5 (6.8%)	
	Mucinous	0	2 (2.7%)	
Grade	1	4 (66.7%)	6 (85.7%)	0.71
	2	1 (16.7%)	1 (14.3%)	
	3	1 (16.7%)	0	
Lymphovascular space involvement		22 (46.8%)	13 (39.4%)	0.51
FIGO Stage	IIB	2 (2.2%)	2 (2.7%)	0.44
	III	74 (83.1%)	67 (89.3%)	
	IV	13 (14.6%)	6 (8%)	
Type of recurrence	Lymph node	7 (16.7%)	6 (17.6%)	0.24
	Peritoneal Carcinomatosis	31 (73.8%)	20 (58.8%)	
	Metastasis	1 (2.4%)	5 (14.7%)	

Table S4: Characteristics of Patients in the advanced-stage group. Time-to-Chemotherapy before and after 8 weeks

Variables		≤8 weeks (*n* = 126)	>8 weeks (*n* = 34)	*p*
Age (years)		60 (+/- 12)	59 (+/- 12)	0.69
BMI (Kg/m2)		24.23 (+/- 4.19)	24.5 (+/- 5.09)	0.75
Hormonal Status	Menopause	93 (78.8%)	26 (74.3%)	0.57
	Menopausal Hormone Therapy	24 (32.4%)	7 (33.3%)	0.94
Parity		1.975 (+/- 1.51)	1.297 (+/- 1.22)	0.014
Mutation	BRCA 1	9 (27.3%)	3 (27.3%)	0.64
	BRCA 2	4 (12.1%)	1 (9.1%)	
Hypertension		19 (20.7%)	8 (27.6%)	0.43
Diabete		4 (4.7%)	2 (7.1%)	0.63
Smoking		4 (5.6%)	2 (9.5%)	0.62
Histological type	Serous	96 (82.8%)	30 (83.3%)	0.45
	Endometrioid	13 (11.2%)	2 (5.6%)	
	Clear Cells	6 (5.2%)	3 (8.3%)	
	Mucinous	1 (0.9%)	1 (2.8%)	
Grade	1	6 (75%)	4 (80%)	1
	2	1 (12.5%)	1 (20%)	
	3	1 (12.5%)	0 (0%)	
lymphovascular space involvement		27 (45.8%)	8 (38.1%)	0.54
FIGO Stage	IIB	4 (3.1%)	0 (0%)	0.82
	III	108 (85%)	33 (89.2%)	
	IV	15 (11.8%)	4 (10.8%)	
Type of recurrence	Lymph node	9 (15.3%)	4 (23.5%)	0.64
	Peritoneal Carcinomatosis	39 (66.1%)	12 (70.6%)	
	Metastasis	6 (10.2%)	0 (0%)	

Table S5: Univariate Cox regression model – Recurrence-free Survival

Variable		HR on RFS	*p*
Time To Chemotherapy (days)		1.03 [0.98-1.09]	0.23
Age of diagnosis (years)		1.01 [0.99-1.02]	0.46
BMI (Kg/m2)		1.01 [0.97-1.06]	0.53
Hormonal Status	Menopause	0.95 [0.6-1.51]	0.83
	Menopausal Hormone Therapy	1.28 [0.75-2.17]	0.37
Parity			
Mutation	BRCA 1	1.38 [0.56-3.39]	0.83
	BRCA 2	1.8 [0.52-6.28]	
Hypertension		1.2 [0.74-1.95]	0.46
Diabetes		1.61 [0.7-3.69]	0.26
Smoking		2.07 [0.88-4.89]	0.09
Histologic type	Serous	1	0.92
	Endometrioid	0.96 [0.42-2.18]	
	Clear Cells	1.45 [0.39-5.33]	
	Mucinous	0.97 [0.5-1.9]	
Grade	1	0.13 [0.01-1.28]	0.087
	2	0.29 [0.02-3.31]	
	3	1	
Lymphovascular space involvement		1.28 [0.68-2.4]	0.45
Stage	Early	1.33 [0.86-2.05]	
	Advanced	1	0.19
FIGO Stage	I		
	II		
	III		
	IV		
Type of recurrence	Lymph node	2.41 [0.8-7.22]	0.35
	Peritoneal Carcinomatosis	2.46 [0.89-6.8]	
	Metastasis	2.24 [0.68-7.4]	

Table S6: Multivariate Cox regression model – Recurrence-free Survival

	HR on RFS	*p*
Time To Chemotherapy	1.03 [0.81-1.32]	0.79
Smoking	2.00 [0.22-18.12]	0.54
Early stages	2.68 [0.64-11.30]	0.1794
Grade 1	0.04 [0-0.72]	0.02891
Grade 2	0.08 [0-1.53]	0.09283
